# Microenvironment Engineering as a Design Principle for Suppressing Catalyst Metal Loss

**DOI:** 10.1002/anie.3661635

**Published:** 2026-07-17

**Authors:** Sumin Lim, Guilherme V. Fortunato, Xiangyu You, Wei Zhao, Marc Ledendecker

**Affiliations:** ^1^ Sustainable Energy Materials Technical University of Munich Campus Straubing Straubing Germany; ^2^ Forschungszentrum Jülich GmbH Helmholtz Institute Erlangen‐Nürnberg For Renewable Energy Erlangen Germany

**Keywords:** catalyst microenvironment, electrocatalyst stability, ionomer–catalyst interactions, *operando* ICP‐MS, platinum dissolution, pore‐confinement

## Abstract

Stability losses in platinum electrocatalysts arise from dissolution, redeposition, and restructuring processes that are strongly shaped by the catalyst microenvironment. Yet the degree to which ionomer chemistry and support porosity jointly govern the true platinum mass balance remains unresolved. Here, by integrating *operando* ICP‐MS with an ion‑exchange approach that quantifies platinum species retained within the ionomer, we distinguish apparent dissolution from actual metal loss across model polycrystalline platinum and supported nanoparticles. We find that direct ionomer‐platinum contact enhances intrinsic dissolution while simultaneously trapping dissolved platinum ions, leading to a pronounced mismatch between measured and actual platinum loss. Extending this framework to porous and nonporous carbon supports reveals that pore‐confinement fundamentally alters degradation pathways, promoting local redeposition and suppressing net metal loss. Our results show that managing ionomer access and nanoscale transport within the catalyst microenvironment is a key practical factor in improving electrocatalyst durability.

## Graphical abstract

Under ionomer‐containing microenvironments, nonporous carbon supports enhance the retention of dissolved platinum ions, whereas porous carbon supports promote pore‐confined redeposition and mitigate net platinum loss.

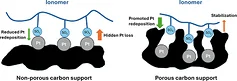

## 1 Introduction

The optimization of electrochemical energy‐conversion systems, such as polymer electrolyte membrane fuel cells (PEM‐FCs) and water electrolyzers (PEM‐WEs), require not only control over elementary surface reactions but also precise tuning of the coupled transport processes spanning multiple length scales. While substantial progress in catalyst development has been driven by binding‐energy descriptors and volcano‐type relationships, the complex, multiscale catalyst‐electrolyte microenvironment remains poorly understood [1, 2, 3, 4]. For kinetic and mass‐transport regimes in PEM fuel cells, an ideal carbon support features internal pores that are accessible enough to enable proton and O_2_ transport yet narrow enough to restrict ionomer penetration, thereby shielding platinum nanoparticles (Pt NPs) from direct ionomer adsorption [5, 6, 7, 8, 9, 10]. Although such microenvironmental considerations are increasingly recognized in performance evaluations [11, 12, 13, 14, 15, 16, 17], their influence on catalyst stability is equally critical but remains largely unexplored. Studies have shown that the combination of sulfonate (─SO_3_^−^) groups, hydrophobic polymer backbones, and confined water domains can accelerate Pt dissolution when the ionomer directly contacts the metal surface [18, 19], whereas another report has suggested an apparent stabilization effect due to reduced diffusion of dissolved Pt species within the ionomer phase [13]. These conflicting observations reveal a fundamental knowledge gap in how ionomer chemistry and support morphology jointly govern catalyst degradation.

In this work, we reconcile these standing inconsistencies by systematically interrogating the Pt‐ionomer interface across both polycrystalline model surfaces and carbon‐supported NPs. By integrating *operando* metal detection with quantitative ion exchange analysis of ionomer‐retained Pt species, we establish a framework that distinguishes transient dissolution from permanent Pt mass loss. We reveal that degradation is not a simple function of potential, but is instead governed by a “dissolution mismatch” dictated by the catalyst microenvironment. While nonporous supports facilitate severe ionomer‐induced Pt loss, porous architectures exploit pore confinement to promote local redeposition and retention of dissolved species. By modulating electrochemical scan rates, we further shed light on the kinetic competition between ion diffusion and redeposition. Ultimately, these insights provide a mechanistic framework for ionomer‐mediated degradation, offering design guidelines for decoupling catalytic activity from material instability.

## 2 Experimental Section

### 2.1 Polycrystalline Pt Bulk and Carbon‐Supported Pt NPs Samples

All experiments were performed in a custom‐made channel flow cell (CFC) operated as a three‐electrode system, in 0.1 M HClO_4_ electrolyte [20]. A commercially available reference electrode (Mini‐Hydroflex, Gaskatel, Germany) and a graphite rod were used as a reference and counter electrode, respectively. A custom‐made polycrystalline Pt (diameter: 5 mm) was employed as the working electrode, embedded into a PEEK body, providing a well‐defined Pt surface without the support‐derived pore‐confinement inherent to carbon‐supported nanoparticle catalysts.

Prior to the addition of the ionomer, the electrochemically active surface area (ECSA_CO_) of the bare Pt bulk and Pt NPs were determined by CO stripping voltammetry to standardize ionomer coverage. CO stripping voltammetry was conducted in 0.1 M HClO_4_ electrolyte to determine the ECSACO for Pt bulk and Pt NPs electrodes, both before and after ionomer addition. While the working electrode was held at 0.06 V_RHE_, the electrolyte in the CFC was saturated with CO gas for 10 min, followed by 10 min of N_2_ purging to remove excess CO, except for adsorbed CO on Pt. Two consecutive cyclic voltammograms (CVs) were then recorded from 0.06 to 1.1 V_RHE_ at 50 mV·s^−1^, with the first for CO stripping and the second serving as the background for ECSA_CO_ calculation.

Although the adsorption of ionomer on Pt bulk differs from the Pt NPs, the ECSA_CO_ normalized ─SO_3_^−^ amount provides a useful comparison of accessible ionomer per electrochemically active Pt site. Nafion (5 wt%, 1100 EW) was diluted 10‐fold in a solvent mixture of 40 vol% isopropanol and Milli‐Q water, and 5 µL of the solvent mixture was drop‐casted onto the mirror‐polished polycrystalline Pt electrode.

For Pt NPs on different carbon supports, commercially available Pt/Vulcan (Pt/V, TEC10V20E, 19.8 wt%, Tanaka, Japan) and Pt/Ketjenblack (Pt/KB, TEC10E20E, 19.4 wt%, Tanaka, Japan) catalysts were dispersed in a solvent mixture of 40 vol% isopropanol/Milli‐Q water. Nafion was then added to the ink at an ionomer‐to‐catalyst (I/C) weight ratio of 0.7. This I/C ratio was selected as an application‐relevant ionomer loading, allowing direct comparison with our previous degradation study [19] using the same Pt/V and Pt/KB catalysts. The inks were homogenized by ultrasonication and drop‐casted onto a custom‐made mirror‐polished glassy carbon electrode (diameter: 5 mm), resulting in a Pt loading of 20 µg_Pt_·cm^−2^.

The added Nafion amounts, normalized to ECSA_CO_, correspond to 2.3 nmol of ─SO_3_^−^ for the Pt bulk, 2.87 nmol of ─SO_3_^−^ for Pt/V, and 2.86 nmol of ─SO_3_^−^ for Pt/KB. This normalization provides a comparable order of magnitude for the ─SO_3_^−^ amount per ECSA_CO_ across electrodes, enabling a direct comparison of microenvironment effects. The details of ECSA_CO_ for all electrodes are provided in Table S1.

### 2.2 Electrochemical Protocol and Potential‐Resolved *Operando* ICP‐MS

To probe the effects of Nafion and support‐induced microenvironments on Pt stability, an identical electrochemical protocol was applied to all electrodes following the initial activation CVs in 0.1 M HClO_4_ until the hydrogen underpotential deposition (H_UPD_) charge no longer increased, ensuring effective surface cleaning and activation. The Pt bulk electrode was activated using 10 cycles of CVs at 200 mV·s^−1^ from 0.06 to 1.4 V_RHE_, while Pt/V and Pt/KB underwent 25 cycles of CVs from 0.06 to 1.1 V_RHE_ at 200 mV·s^−1^.

After activation, the protocol consisted of a 3‐min anodic hold at 1.1 V_RHE_ to promote Pt oxide formation and initiate place‐exchange‐driven transient dissolution [21]. This was followed by a cathodic scan from 1.1 V_RHE_ to 0.06 V_RHE_ at slow (10 mV·s^−1^) and fast (100 mV·s^−1^) scan rates, which induced substantial Pt dissolution during the reduction of Pt oxide to metallic Pt [20, 22, 23]. The slow cathodic scan prolongs diffusion time for dissolved Pt ions, thereby minimizing redeposition, whereas the fast cathodic scan reduces diffusion time, increasing the probability of redeposition on Pt or carbon supports [21, 24]. All measurements were conducted in an in‐house designed CFC coupled to ICP‐MS (NexION 2000B, PerkinElmer) for real‐time quantification of dissolved Pt ions. The 0.1 M HClO_4_ electrolyte was pumped at a rate of 0.25 mL·min^−1^ into the ICP‐MS nebulizer. To ensure steady ICP‐MS performance, calibration tests were conducted using a ^187^Re internal standard (10 ppb in 0.1 M HClO_4_, selected for its mass and ionization characteristics similar to those of Pt), which was introduced downstream during calibration and measurements.

### 2.3 Ion Exchange

Ex‐situ La^3+^–Pt^2+^ ion exchange was performed on all electrodes to displace Pt species electrostatically or coordinatively associated with ─SO_3_^−^ groups and to quantify the fraction of dissolved Pt entrapped within Nafion. La^3+^ was selected as an exchanging cation because the trivalent cations exhibit stronger adsorption to ─SO_3_^−^ groups in Nafion compared to platinum and can effectively replace lower valency cations, while its low mobility in Nafion minimizes back transport during the exchange step [25, 26, 27, 28]. A 0.1 M La(ClO_4_)_3_ solution was prepared by dissolving lanthanum perchlorate hexahydrate (La(ClO_4_)_3_·6H_2_O, Fisher Scientific) in Milli‐Q water/HClO_4_, resulting in a pH 1 to maintain an acidic environment and suppress the precipitation of Pt ions during the exchange procedure. After the electrochemical protocol, the electrodes were immersed in 0.1 M La(ClO_4_)_3_ solution at 70°C for 21 h to promote the ion exchange step within Nafion. This experimental condition was selected based on time‐dependent ion‐exchange experiments performed for Pt/V and Pt/KB using the same electrochemical protocol, with exchange times of 3, 6, 21, and 48. The resulting solutions were analyzed by ICP‐MS to quantify the exchanged Pt^2+^ ions (m_IE_). For Pt bulk, residual Nafion was mechanically collected using a filter paper (Whatman 541, Merck). The filter paper was subsequently digested in aqua regia and analyzed by ICP‐MS (m_AR_) to quantify the amount of remaining Pt in Nafion. This value was used to estimate the ion exchange efficiency (*η*) for all electrodes. Prior to ICP‐MS analysis, 0.1 M La(ClO_4_)_3_ extracts were diluted 500‐fold in 0.1 M HClO_4_, and digested filter papers in aqua regia were diluted 100‐fold in ultrapure water to prevent matrix overloading. The corresponding blank solutions were measured and subtracted. The ion exchange efficiency, *η*, was determined using Equation (1), defined as the mass of Pt ions exchanged by La^3+^ (m_IE_​) relative to the total Pt mass (m_IE_+m_AR_) recovered in the Nafion fraction for the bulk Pt electrode.

(1) $$\eta \left(\%\right)=\frac{m_{\text{IE}}}{m_{\text{IE}}+m_{\text{AR}}}\times 100$$

## 3 Results and Discussion

### 3.1 CFC‐ICP‐MS for Pt Bulk Sample

**FIGURE 1:**
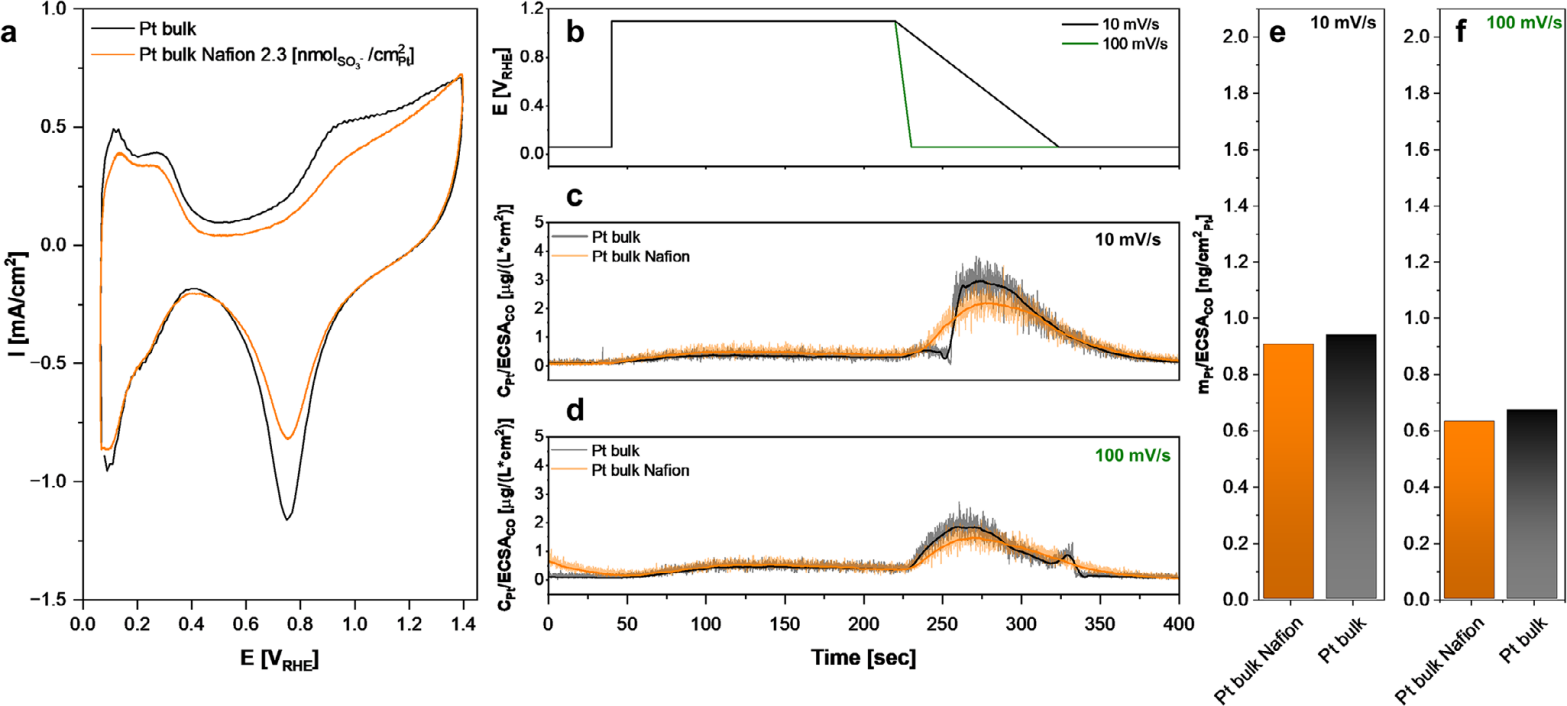
(a) CVs of a polycrystalline Pt electrode (geometric area = 0.196 cm^2^) without Nafion (black) and with Nafion (orange). The Nafion loading is expressed as the concentration of ─SO_3_^−^ groups normalized to the ECSA_CO, Bare_. (b) Applied electrochemical protocol consisting of a potential hold at 1.1 V_RHE_ for 3 min, followed by a cathodic potential scan at 10 mV·s^−1^(black) or 100 mV·s^−1^ (green). Time‐resolved Pt dissolution profiles normalized to ECSA_CO_ corresponding to cathodic scan rates of (c) 10 mV·s^−1^ and (d) 100 mV·s^−1^, respectively. Amount of dissolved Pt normalized to ECSA_CO_ for scan rates of (e) 10 mV·s^−1^ and (f) 100 mV·s^−1^.

To establish a mechanistic framework for assessing catalyst stability within its microenvironment, we first examined Pt as a benchmark system using a well‐defined polycrystalline Pt surface. We quantified potential‐dependent dissolution in the presence of controlled amounts of ─SO_3_^−^ groups while independently determining the total irreversible Pt loss. Geometry‐normalized voltammograms of Pt bulk revealed that Nafion substantially suppresses both H_UPD_ features and the charges associated with oxide formation and reduction, reflecting the specific adsorption of ─SO_3_^−^ groups that block Pt sites, perturb proton adsorption, and compete with oxygenated adsorbates generated during oxide growth [29, 30]. Thus, the decrease in H_UPD_ and Pt oxide formation/reduction charges in Figure 1a indicates a reduced electrochemically available Pt surface area within the modified Pt/Nafion microenvironment. Figure 1b displays the electrochemical protocol, along with the corresponding ECSA_CO_‐normalized potential‐resolved *operando* ICP‐MS Pt dissolution profiles (Figure 1c,d) recorded with the cathodic scans at both 10 and 100 mV·s^−1^. Varying the cathodic scan rate alters the timescales of redeposition. It modulates how long dissolved Pt ions reside near the surface before the potential reaches the redeposition regime. Consequently, a slow cathodic scan favors Pt ion diffusion through the ionomer film, whereas a fast cathodic scan preferentially promotes rapid electrochemical redeposition, resulting in less measured Pt dissolution. In the presence of Nafion, the absolute Pt dissolution decreased relative to the bare Pt bulk. Normalization against ECSA_CO_ revealed similar Pt dissolution amounts for both electrodes with and without Nafion. Consequently, the apparent suppression of dissolution is likely attributed to a loss of electrochemically accessible Pt surface area instead of improved intrinsic stability. The slightly lower ECSA_CO_ normalized dissolution observed with Nafion compared to the bare electrode (for both scan rates in Figure 1e,f, respectively) suggests that a fraction of the dissolved Pt may not have been released into the electrolyte but instead trapped by the ─SO_3_^−^ groups, as only Pt ions transported out of the catalyst layer into the electrolyte are monitored. Hence, these results are consistent with a combination of (1) reduced accessible Pt sites, (2) redeposition of Pt ions to metallic Pt, and (3) possible Pt entrapment by ─SO_3_^−^ groups. Similar behaviors have been reported in membrane electrode assemblies (MEA), where dissolved Pt ions accumulated within the membrane, forming a Pt band after reduction from H_2_ crossover from the anode [31, 32, 33].

**FIGURE 2:**
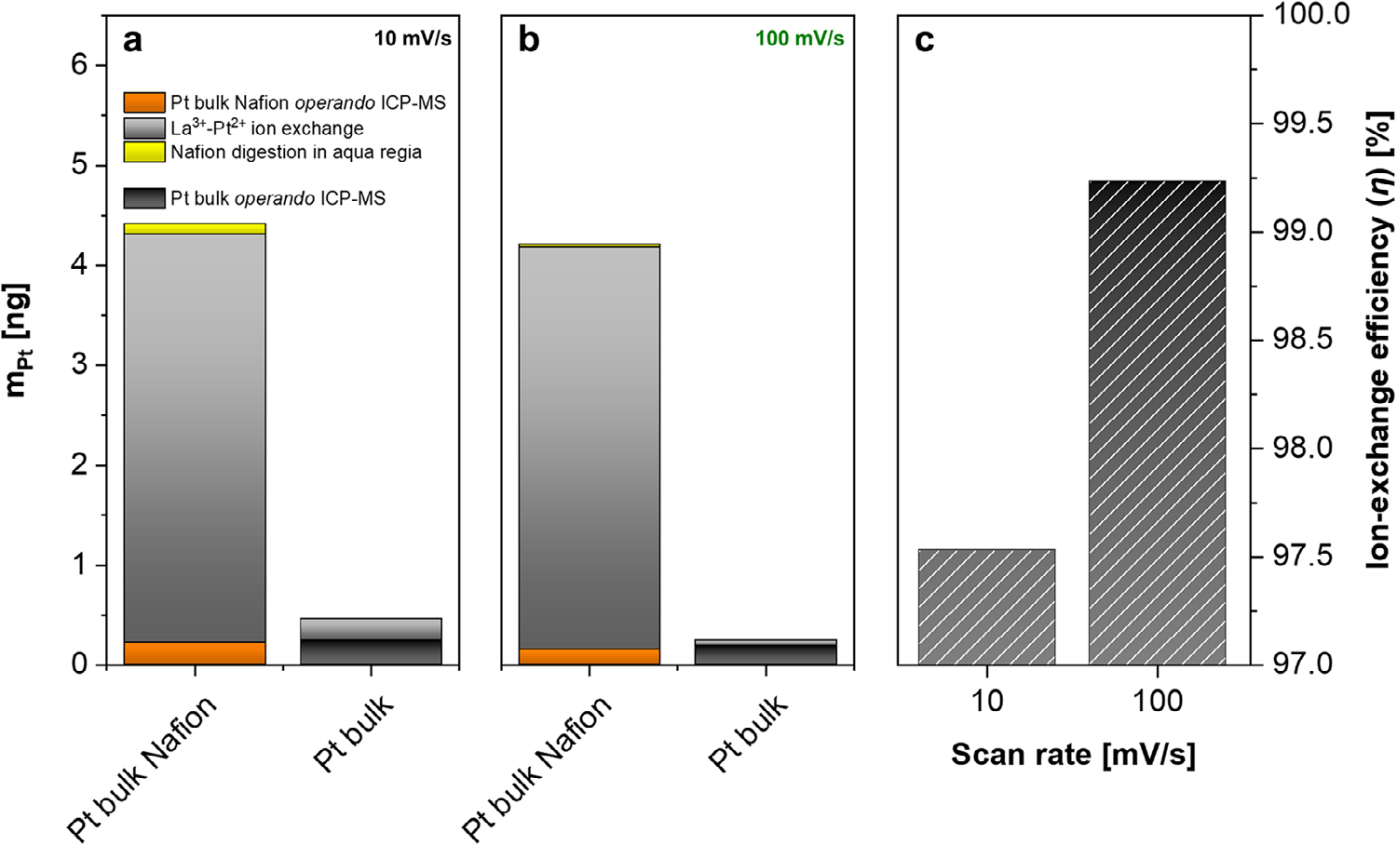
Amount of dissolved Pt quantified by *operando* ICP‐MS (orange), ion exchange analysis (grey), and post‐experiment aqua regia digestion (yellow) at cathodic scan rates of (a) 10 mV·s^−1^, and (b) 100 mV·s^−1^. (c) *Operando* exchange efficiency (*η*) calculated according to Equation (1).

Direct comparison between Pt bulk with and without Nafion reveals that Pt dissolution in potential‐resolved *operando* ICP‐MS is of the same order of magnitude in all electrodes. To quantify the entrapped Pt within Nafion and thus invisible to *operando* ICP‐MS, we performed La^3+^–Pt^2+^ ion exchange, where ion exchange reveals an order‐of‐magnitude higher amount of Pt than *operando* ICP‐MS (Figure 2a,b). Specifically, total Pt loss in the presence of Nafion was nearly 10 times higher than the bare Pt bulk at 10 mV·s^−1^ and 17 times higher at 100 mV·s^−1^. The high La^3+^–Pt^2+^ ion exchange efficiencies in Figure 2c (97.5% at 10 mV·s^−1^ and 99.2% at 100 mV·s^−1^) confirm effective exchange of trapped Pt ions. The shortened diffusion time at 100 mV·s^−1^ may allow a larger fraction of dissolved Pt ions to be redeposited on the Pt surface, resulting in slightly higher exchange efficiency. Fast cathodic scan rate primarily reduces Pt dissolution on bare Pt bulk by promoting redeposition, whereas Nafion traps a substantial fraction of dissolved Pt ions, largely independent of the scan rate due to direct Nafion‐Pt contact and rapid entrapment of Pt ions to ─SO_3_^−^. Although the ion exchange was performed without external polarization, additional Pt dissolution during the open circuit cannot be excluded [34]. Consequently, the mass of Pt ions exchanged by La^3+^ (m_IE_) represents an upper bound for the Pt entrapped within the Nafion layer. This quantification of “unaccounted” Pt loss provides a rigorous basis for previous reports suggesting that Nafion exacerbates ECSA loss and accelerates Pt coarsening on nonporous carbon‐supported catalysts. Our findings support the hypothesis that extensive ─SO_3_^−^ adsorption destabilizes exposed Pt surfaces, thereby facilitating the dissolution and subsequent coarsening observed in these systems [19, 35].

### 3.2 CFC‐ICP‐MS for Carbon‐Supported Pt NPs Samples

**FIGURE 3:**
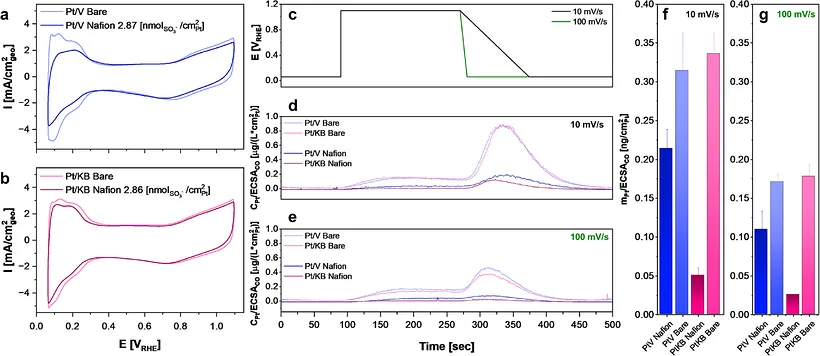
CVs normalized to the geometric electrode area (0.196 cm^2^) of (a) Pt/V and (b) Pt/KB catalysts. The Nafion loading is expressed as the concentration of ─SO_3_^−^ groups per ECSA_CO, Bare_. (c) Applied electrochemical protocol consisting of a potential holding at 1.1 V_RHE_ for 3 min, followed by a cathodic scan rate of 10 mV·s^−1^(black line), and 100 mV·s^−1^(green line). Time‐resolved Pt dissolution profiles normalized to ECSA_CO_ corresponding to scan rates of (d) 10 mV·s^−1^ and (e) 100 mV·s^−1^, respectively. Total amount of dissolved Pt normalized to ECSA_CO_ for scan rates of (f) 10 mV·s^−1^ and (g) 100 mV·s^−1^. Error bars represent the standard deviation from at least three independent measurements.

To extend the insights from Pt bulk model electrodes to supported structures on carbon, we investigated a nonporous carbon material, where Pt NPs are located on the outside of the primary carbon particles, and a porous carbon support, where Pt NPs are mainly located inside (and partially outside) the mesoporous structure. According to the scanning transmission electron microscopy (STEM) analysis reported in our previous study [19], approximately 35% of the counted Pt particles in pristine Pt/V were externally visible on the outer carbon support/aggregate, whereas only approximately 5% were externally visible in pristine Pt/KB. For better comparison with polycrystalline Pt bulk, the same electrochemical protocol was applied. The CVs of Pt/V, Pt/KB, normalized to geometric electrode area, are shown in Figure 3a,b. In the presence of Nafion, both catalysts exhibited reduced H_UPD_ and CO stripping charge, consistent with a reduction in electrochemically accessible Pt sites observed in polycrystalline Pt.

The more significant ECSA decrease observed for Pt/V compared to Pt/KB likely arises from enhanced adsorption of Nafion ─SO_3_^−^ groups on Pt NPs supported on nonporous carbon (c.f. Figure S1). Previous studies have shown that the ionomer preferentially wets external carbon surfaces and forms a more extensive coverage of ionomer on nonporous carbon supports, whereas porous carbon supports often require a relatively higher ionomer content to achieve comparable ionomer coverage on Pt due to incomplete pore filling [36, 37, 38, 39]. This spatial separation indicates that Pt dissolution in Pt/KB likely involves a larger contribution from the Pt NPs in the pore, where the local ionomer morphology, hydration, and Pt ion transport resistance can differ from those at the outer surface. In this regard, we describe the porous support effect in terms of pore‐confined mass transport, where Pt NPs within or near the pore may remain less directly contacted with the ionomer while dissolved Pt ions experience less probability of direct escape into the bulk electrolyte. In our previous study on Pt/V and Pt/KB, identical‐location STEM analyses were used to track identical catalyst locations before and after 10 000 accelerated degradation between 0.6 and 1 V_RHE_, potential hold of 1s [19]. These measurements showed the disappearance of small Pt NPs, accompanied by the growth of the remaining particles, providing morphological evidence for Pt redistribution via dissolution, followed by redeposition and/or coarsening. These location‐dependent morphological changes are consistent with a pore‐confined microenvironment, in which dissolved Pt ions have a reduced probability of escaping directly into the bulk electrolyte and undergo local redeposition within the pores, thereby leading to higher retention of small Pt particles under confined conditions.

Figure 3c depicts the anodic holding at 1.1 V_RHE_ followed by two cathodic scan rates of 10 and 100 mV·s^−1^. Figure 3d,e shows the resulting ECSA_CO_ normalized Pt dissolution profile in *operando* ICP‐MS at each scan rate. We note that Pt ion adsorption on carbon cannot be fully excluded, especially for high surface area of KB. However, the ECSA_CO_ normalized Pt dissolution of Pt/V and Pt/KB without Nafion was similar under identical experimental conditions. This comparison indicates that differences in carbon surface area and intrinsic carbon support chemistry alone are unlikely to be the dominant origin of a pronounced difference in ECSA_CO_ normalized Pt dissolution. If Pt ion adsorption on the high surface area KB were the dominant mechanism, a stronger suppression of the dissolved amount of Pt during *operando* ICP‐MS would be expected for bare Pt/KB. Therefore, the mismatch observed upon Nafion addition is more consistently attributed to differences in effective ionomer accessibility, pore‐confined Pt ion transport, and redeposition probability. Nevertheless, a slightly higher absolute Pt dissolution was observed for Pt/KB, which is in line with the slightly smaller particle size as analyzed with STEM [19]. Although the average particle size of Pt/KB (2.4 ± 0.7 nm) is smaller than Pt/V (2.7 ± 1.2 nm), the ECSA_CO_ of Pt/KB (88.9 ± 0.36 m^2^·g^−1^) was in the similar range of Pt/V (88.71 ± 3.87 m^2^·g^−1^), possibly due to the underestimated accessible Pt surface area in the porous support, as reported in a previous study [40]. As shown in Figure 3f,g, the presence of Nafion induces a pronounced support dependence in the ECSA_CO_ normalized *operando* Pt dissolution, even though both catalysts exhibit lower overall dissolution than their Nafion‑free counterparts. For all electrodes, regardless of the presence of Nafion, the high cathodic scan rate reduced the amount of dissolved Pt by a consistent fraction, approximately 45%–50% when normalized to ECSA_CO_. This suggests that the electrochemical timescale remains a dominant parameter governing dissolution kinetics across these systems. Upon Nafion addition, Pt/KB exhibits an approximately four‐fold lower amount of dissolved Pt than Pt/V at both scan rates. This support‐dependent suppression is not readily explained by the electrochemical protocol alone and may reflect the pore‐confinement effect in the microenvironment discussed above. In this context, the reduced *operando* dissolution signal on Pt/KB with Nafion should be interpreted as a lower escape probability of dissolved Pt ions from pore‐confined microenvironment rather than as direct evidence of intrinsic stabilization.

**FIGURE 4:**
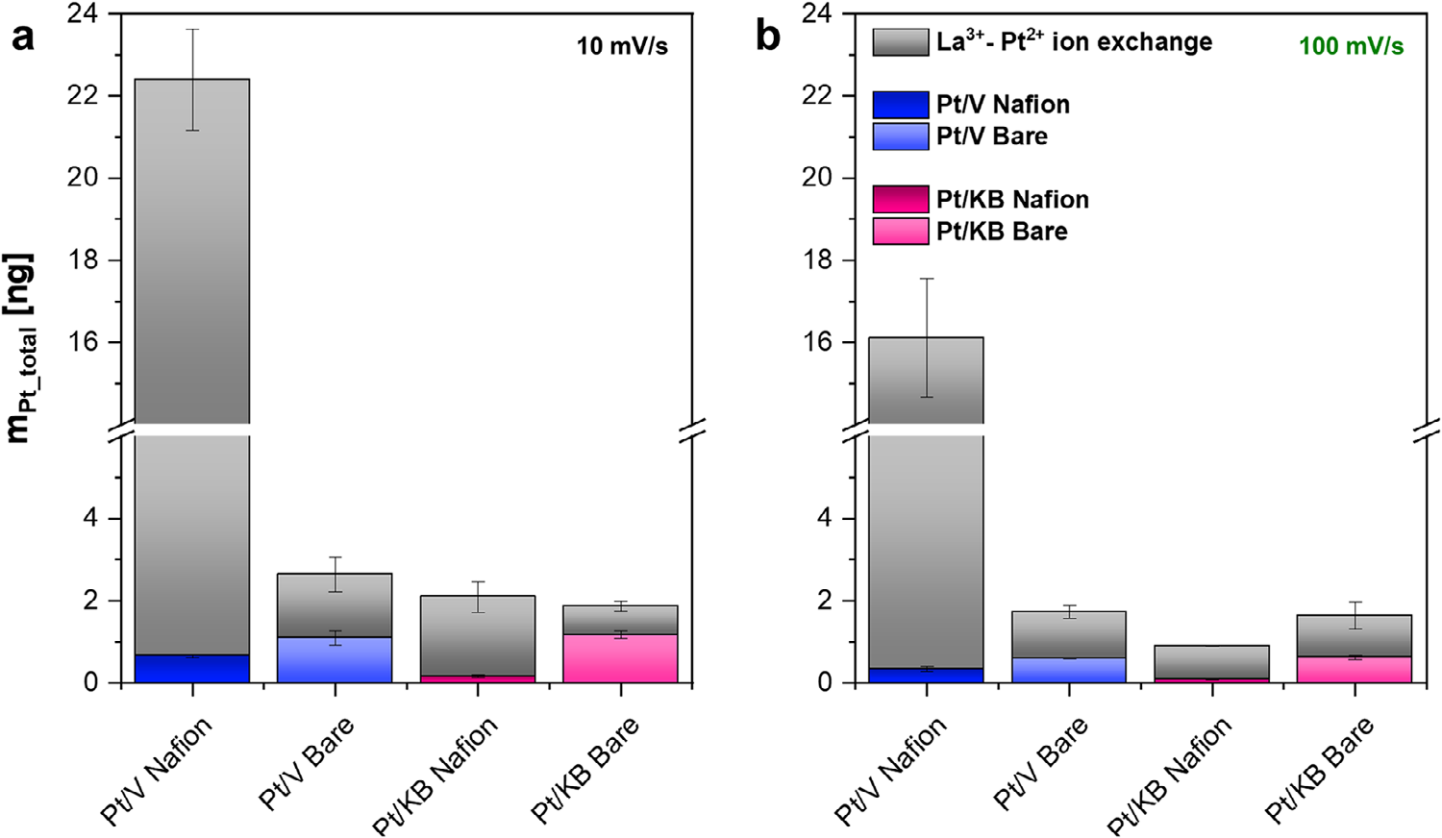
Amount of dissolved Pt quantified from *operando* ICP‐MS (blue and red for Pt/V, and Pt/KB respectively), ion exchange analysis (grey) at the cathodic scan rate of (a) 10 mV·s^−1^, and (b) 100 mV·s^−1^. All measurements were represented with an error bar from at least three repeated measurements.

To quantitatively assess the total irreversible Pt loss beyond the dissolved Pt detected by *operando* ICP‐MS for Pt/V and Pt/KB, which is influenced by Nafion‐Pt interactions, La^3+^–Pt^2+^ ion exchange was performed, and the resulting total amount of Pt loss is presented in Figure 4a,b. Unlike in the case of bulk Pt, selective digestion of Nafion in aqua regia could not be applied to Pt NPs because the ionomer cannot be separated from the catalyst layer without affecting the Pt NPs.

Therefore, the ion exchange measurements for Pt/V and Pt/KB represent the exchangeable trapped Pt ion under standardized exchange conditions. To assess whether La^3+^ diffusion through the catalyst layer limits ion exchange, we performed time‐dependent ion exchange measurements for Pt/V and Pt/KB at a 100 mV·s^−1^ cathodic scan rate. The La^3+^ exchangeable Pt amount increased with exchange time and approached a plateau after the 21 h exchange time used in this study (Figure S2), indicating that the selected condition provides sufficient access to trapped Pt for both supports. Therefore, the detected Pt amount from the ion exchange reflects not only the total number of ‐SO_3_^−^ groups added to the electrode, but also their effective accessibility to dissolved Pt ions within the local catalyst microenvironment. For the ion exchange conditions (0.1 M La(ClO_4_)_3_, 70°C for 21 h), using an effective diffusion coefficient of La^3+^ in Nafion (D, 3.50 * 10^−7^ cm^2^·s^−1^) [26], the corresponding diffusion length (L) is approximately 1.63 mm, as shown in Equation (2), which is far larger than the nanometer scale catalyst/ionomer layer. Thus, exchangeable Pt amounts are mainly attributed to support‐dependent Pt ion entrapment within the microenvironment, rather than insufficient La^3+^ access under the selected ion exchange conditions.

(2) $$L=\sqrt{D\times T}=\sqrt{3.50\cdot 10^{-7}\left[{cm}^{2}\cdot s^{-1}\right]\times 75600\left[s\right]}\approx 1.63mm$$

At the slower scan rate of 10 mV·s^−1^, both catalysts exhibited a substantially higher total Pt loss than measured by *operando* ICP‐MS alone, indicating that a significant fraction of dissolved Pt ions was trapped within the Nafion phase rather than being released into the bulk electrolyte. In the presence of Nafion, Pt/V showed a pronounced increase in total Pt loss compared to the bare electrode, consistent with a high ‐SO_3_^−^‐ Pt contact fraction on the nonporous support. In contrast, Pt/KB exhibited only a modest increase in total Pt loss upon Nafion addition, comparable to the bare case, suggesting that the porous carbon geometry partially limits Nafion accessibility. When the scan rate is increased to 100 mV·s^−1^, the influence of the carbon support becomes more pronounced. Both catalysts exhibited reduced total Pt loss, consistent with shorter diffusion times for Pt ions to escape into the electrolyte and a higher probability of redeposition. For Pt/V, the presence of Nafion continued to yield higher total Pt loss compared to the bare electrode, indicating that although redeposition is enhanced at faster scan rates, it cannot fully counteract the Nafion‐induced microenvironment that promotes Pt ion trapping. Conversely, Pt/KB exhibited a lower overall Pt loss when Nafion was present compared to the Nafion‑free electrode. This behavior is likely related to the physical separation between Pt particles and Nafion within the porous carbon matrix. When Pt dissolves from sites that are not in direct contact with Nafion, the resulting ions must diffuse through the porous carbon network before reaching the ionomer phase, where they can be electrostatically trapped. Under fast cathodic scan conditions, the combination of a shortened mass‐transport window and the diffusion path out of the pore network significantly increases the local Pt ion concentration. This localized enrichment triggers a Nernstian shift in redeposition potential, thermodynamically favoring the recovery of dissolved Pt species before they can escape the confined environment. If the pore geometry imposes additional transport resistance, or if dissolved Pt ions are generated within the pores with limited direct ionomer accessibility, these ions are less likely to reach the bulk electrolyte and therefore contribute less to the *operando* ICP‐MS signal. Consequently, Pt/KB shows a lower total Pt loss in the presence of Nafion at fast cathodic scan rates, which may be consistent with a microenvironment where confinement‑enhanced redeposition partially competes with, or surpasses, Pt entrapment within Nafion.

**FIGURE 5:**
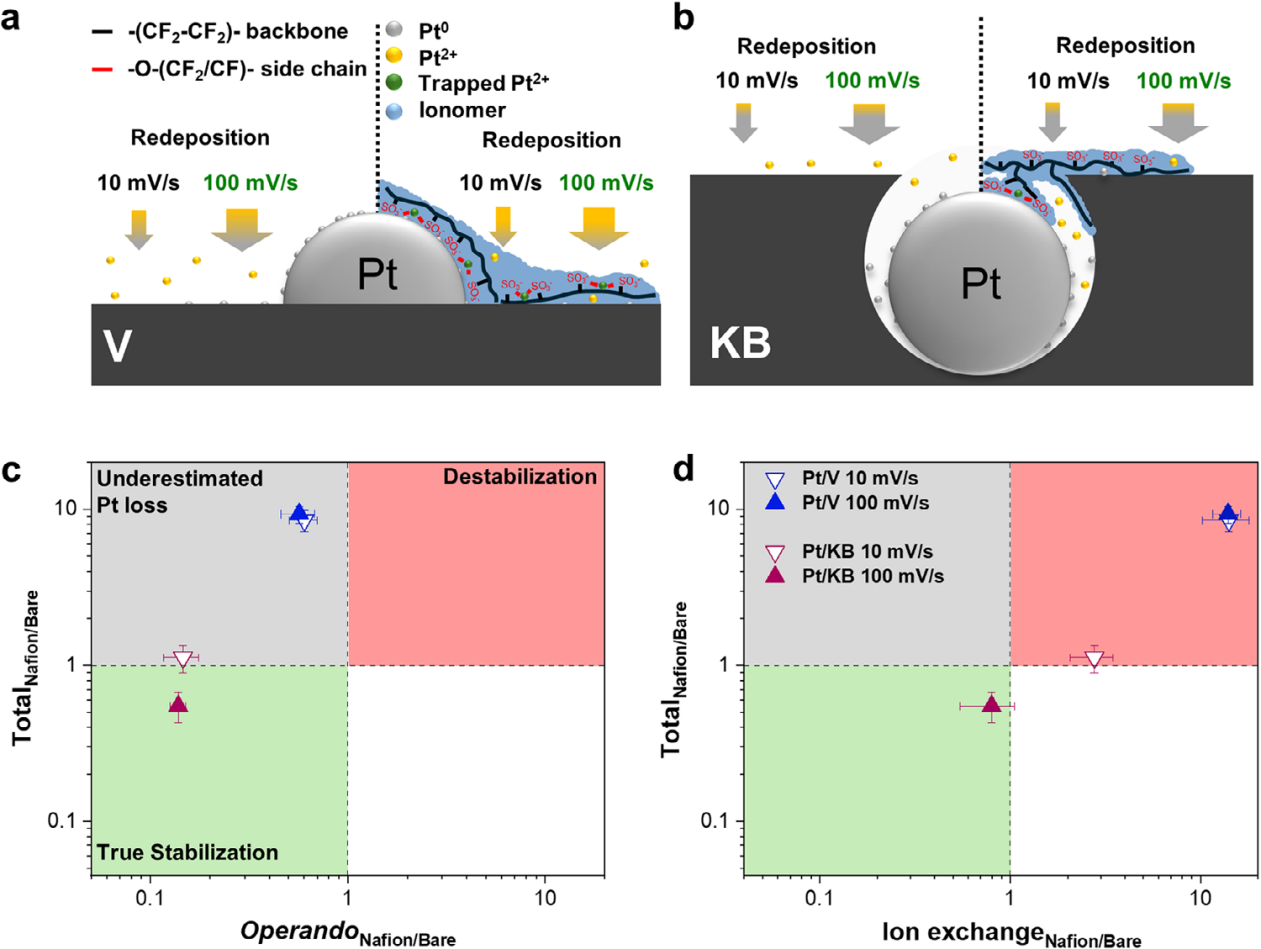
Schematic representation of the proposed microenvironment separated by the black dotted line for the absence of Nafion (left) and presence of Nafion (right). The degree of redeposition is illustrated as color gradient for (a) Pt/V, and (b) Pt/KB. (c) Quadrant map defined by measured apparent Pt mass loss, m*_operando_*_,Nafion_/m*_operando_*_, Bare_ (*x*‐axis), and total Pt mass loss m_total,Nafion_/m_total, Bare_ (*y*‐axis). (d) Quadrant map defined by measured hidden Pt mass loss, m_IE,Nafion_/m_IE, Bare_ (*x*‐axis), and total Pt mass loss m_total,Nafion_/m_total, Bare_ (*y*‐axis), highlighting the relative contribution of Nafion trapped Pt to the net Pt loss.

Figure 5a,b schematically illustrates the ionomer‐induced microenvironment for the Pt/V and Pt/KB catalysts. Figure 5c,d presents these results as quadrant plots that relate total Pt loss to *operando* and ion exchange‐retained Pt dissolution, distinguishing true stabilization (first quadrant), underestimated Pt loss (second quadrant), and destabilization (third quadrant). In Figure 5c, the *x*‐axis shows the influence of Nafion in Pt dissolution as a ratio (*Operando*_Nafion/Bare_) in *operando* ICP‐MS measurements. The *y*‐axis shows the influence of Nafion in total Pt loss as a ratio (Total_Nafion/Bare_), which reflects the net Pt loss from the electrode.

The third quadrant, where both ratios are below unity, is assigned to conditions where a reduced amount of *operando* Pt dissolution is accompanied by a reduced total Pt loss. Notably, only Pt/KB at a fast cathodic scan rate is placed in this third quadrant regime, due to the limited Nafion accessibility and favored redeposition. In contrast, the second quadrant, where the *Operando*_Nafion/Bare_ < 1, while Total_Nafion/Bare_ > 1, indicates that the reduced amount of *operando* Pt dissolution does not translate into a lower total Pt loss, consistent with a larger fraction of dissolved Pt being trapped within the Nafion phase and remaining invisible to *operando* ICP‐MS. The extent of the underestimation is clarified in Figure 5d by replacing the *x*‐axis with the ratio of trapped Pt ion masses in the electrodes between the Nafion containing one and the bare (Ion exchange_Nafion/Bare_), while keeping the same *y*‐axis. The conditions that appeared below unity only for the ratio of *Operando*_Nafion/Bare_ in Figure 5c are shifted to above the unity in Figure 5d, showing that the hidden Pt loss is recovered as an increased Nafion‐associated loss by ion exchange. Meanwhile, Pt/KB at fast cathodic scan rate remains in the third quadrant regime, indicating that both the Nafion‐associated fraction and the total Pt loss are reduced under a spatially isolated microenvironment. The first quadrant, where both ratios exceed unity, represents conditions under which Nafion increases both the Pt dissolution from *operando* and the total Pt loss. Accordingly, our results suggest that catalyst stabilization depends heavily on a support‐mediated microenvironment that reduces the probability of direct ionomer‐catalyst interaction while favoring local redeposition. Since this study utilizes aqueous‐phase electrochemical analysis, these findings establish a mechanistic framework for Nafion‐based, ionomer‐interfaced electrocatalysis, including CO_2_ reduction or water electrolysis, in which the support architecture dictates the local concentration gradients of ─SO_3_^−^ groups that govern degradation. Considering the rapid potential transients typical of dynamic device operation, porous supports with limited ionomer accessibility can further shift the balance toward lower metal entrapment and higher redeposition rates. Consequently, engineering supports morphology to harness confinement effects emerges as a promising design strategy to mitigate catalyst loss, as the carbon framework can partially shield the Pt from deleterious ionomer interactions.

## 4 Conclusion

This study elucidates how support architecture and ionomer–catalyst interactions shape the fate of dissolved Pt during controlled electrochemical transients. By comparing nonporous and porous carbon supported Pt catalysts, we demonstrate that Nafion's influence is primarily defined by its physical accessibility to the catalyst surface. On nonporous Pt/V, the lack of protective support geometry allows intimate Nafion‐Pt contact, promoting the trapping of Pt ions within the ionomer phase and thereby suppressing the redeposition pathway and increasing net Pt loss. In contrast, the porous structure of Pt/KB acts as a pore‐confined transport environment in which Pt particles in the pore have limited ionomer accessibility. Within these pores, restricted mass transport increases the local concentration of Pt ions, thereby favoring local redeposition. Under fast potential sweeps, this support‐enhanced redeposition can compete with ionomer‐mediated trapping, resulting in a significantly reduced total Pt loss. Collectively, these findings highlight that Pt stability in ionomer‐containing environments is governed by the coupled effects of support morphology, effective ionomer accessibility, Pt‐ion escape probability, and redeposition. Although this study focuses on Nafion as a benchmark sulfonate‐containing ionomer, systematic investigations using alternative ionomers and non‐sulfonated polymers will be an important next step to distinguish sulfonate‐specific Pt ion retention from general polymer film effects. Designing catalyst architectures that leverage support morphology may offer a promising pathway to improve the durability of electrocatalysts in Nafion‐ or sulfonate‐containing microenvironments across a wide range of electrochemical energy conversion technologies.

## Author Contributions

**Sumin Lim**: conceptualization, writing – original draft, methodology, validation, visualization, writing – review and editing, formal analysis, and data curation. **Guilherme V. Fortunato**: conceptualization, writing – review and editing, and supervision. **Xiangyu You**: methodology. **Wei Zhao**: methodology. **Marc Ledendecker**: supervision, resources, project administration, investigation, and funding acquisition.

## Conflicts of Interest

The authors declare no conflicts of interest.

## Supporting information




**Supporting File**: anie73596‐sup‐0001‐SuppMat.docx.

## Data Availability

The data that support the findings of this study are available from the corresponding author upon reasonable request.
