# Supplementary material for: Microenvironment Engineering as a Design Principle for Suppressing Catalyst Metal Loss
Source: Angew Chem Int Ed Engl. 2026 Jul 17;65(38):e3661635. doi: 10.1002/anie.3661635 (PMC13573046; doi:10.1002/anie.3661635)
Supplement: Supplementary file 1 [file ANIE-65-e3661635-s001.docx]

**Supporting Information**

Microenvironment Engineering as a Design Principle for Suppressing Catalyst Metal Loss

**Electrochemical surface area measurements**

The ECSA_CO_ values were obtained by dividing the measured charge (*Q_CO_*) by the charge density (*σ*) of polycrystalline Pt. Q_CO_ was determined by integrating the oxidation peak of CO within the potential ranges of 0.6-1.0 V_RHE_ and 0.7-1.05 V_RHE_ for Pt bulk, Pt/V, and Pt/KB, respectively, as shown in eq S1. CO stripping and background CVs were recorded in 0.1 M HClO_4_ at room temperature, *ν* = 25 mV·s^-1^ (Pt bulk), 50 mV·s^-1^ (Pt/V, Pt/KB). Considering the *σ* as 420 µC·cm^-2^, the ECSA_CO_ values were calculated using eq S2 and presented in Table S1. Additionally, the ECSA_CO_ normalized to the Pt loading (3.92 µg) is presented for Pt/V and KB, in units of m^2^·g^-1^_Pt_. The CO stripping peaks and corresponding background CVs are shown in Figure S1 as current densities normalized to the geometric area (0.19635 cm^2^).

$$\begin{aligned} Q_{\mathrm{CO}}=\frac{\int_{E_{1}}^{E_{2}} (i_{CO}-i_{background})*dE}{\nu}[\mu C]\#\left( eq S1 \right) \end{aligned}$$

$$\begin{aligned} {ECSA}_{CO}=\frac{Q_{CO}}{\sigma} \left[ {cm}_{Pt}^{2} \right]\#\left( eq S2 \right) \end{aligned}$$

**Table S 1**. ECSA values from CO stripping for all repeats as the total surface area in cm^2^_Pt_, with mass normalized values for Pt/V, KB in m^2^·g^-1^_Pt_.

|  | Without Nafion [cm^2^_Pt_] (m^2^·g^-1^_Pt_) | | | | With Nafion [cm^2^_Pt_] (m^2^·g^-1^_Pt_) | | | |
| --- | --- | --- | --- | --- | --- | --- | --- | --- |
| Repeats | 1st | 2nd | 3rd | Error | 1st | 2nd | 3rd | Error |
| Pt bulk | 0.271 | | | - | 0.245 | | | - |
| Pt/V | 3.542 (90.2) | 3.713 (94.56) | 3.195 (81.38) | 0.152 (3.876) | 3.583 (80.65) | 2.492 (91.26) | 3.167 (63.47) | 0.318 (8.09) |
| Pt/KB | 3.479 (88.59) | 3.475 (88.5) | 3.519 (89.62) | 0.014 (0.36) | 3.487 (88.81) | 3.139 (79.93) | 3.559 (90.63) | 0.13 (3.31) |

**Figure S 1**. CO stripping peaks (solid lines) and following background CVs (dotted lines) of Pt disk, Pt/V, and Pt/KB. The current densities were normalized to electrode geometric area (0.19635 cm^2^).

**Figure S 2**. Time-dependent La^3+^ ion exchange profiles of Pt/V (blue) and Pt/KB (red) with the identical electrochemical protocols consisting of a potential hold at 1.1 V_RHE_ for 3 min, followed by a cathodic scan to 0.06 V_RHE_ at a scan rate of 100 mV·s^-1^. The exchanged Pt amount was measured after La^3+^ ion exchange for 3, 6, 21 (used in the main experiments), and 48 h, respectively. All measurements were repeated at least two times individually.
